# Renin-Angiotensin System in Huntington′s Disease: Evidence from Animal Models and Human Patients

**DOI:** 10.3390/ijms23147686

**Published:** 2022-07-12

**Authors:** Lucas M. Kangussu, Natalia P. Rocha, Priscila A. C. Valadão, Thatiane C. G. Machado, Kívia B. Soares, Julliane V. Joviano-Santos, Leigh B. Latham, Gabriela D. Colpo, Ana Flávia Almeida-Santos, Erin Furr Stimming, Ana Cristina Simões e Silva, Antônio L. Teixeira, Aline Silva Miranda, Cristina Guatimosim

**Affiliations:** 1 Department of Morphology, Biological Science Institute, Federal University of Minas Gerais, Belo Horizonte 31270-901, MG, Brazil; lucaskangussu@gmail.com (L.M.K.); drapriscilavaladao@gmail.com (P.A.C.V.); thatacristina01@gmail.com (T.C.G.M.); kiviasoaress@gmail.com (K.B.S.); jullianejoviano@hotmail.com (J.V.J.-S.); pricaufmg@yahoo.com.br (A.F.A.-S.); mirandaas@icb.ufmg.br (A.S.M.); cguatimosim@gmail.com (C.G.); 2 Department of Neurology, The Mitchell Center for Alzheimer′s Disease and Related Brain Disorders, The University of Texas Health Science Center, Houston, TX 77030, USA; natalia.pessoarocha@uth.tmc.edu; 3 McGovern Medical School, HDSA Center of Excellence at The University of Texas Health Science Center, Houston, TX 77030, USA; lcurtis9021@aol.com (L.B.L.); erin.e.furr@uth.tmc.edu (E.F.S.); 4 Neuropsychiatry Program, Department of Psychiatry and Behavioral Sciences, McGovern Medical School, University of Texas, Houston, TX 77054, USA; gabriela.d.colpo@uth.tmc.edu; 5 Center for Mathematics, Computing, and Cognition (CMCC), Federal University of ABC (UFABC), São Bernardo do Campo 09612-000, SP, Brazil; 6 School of Medicine, University of Washington, Seattle, WA 98195, USA; 7 Department of Pediatrics, School of Medicine, Federal University of Minas Gerais, Belo Horizonte 30130-100, MG, Brazil; acssilva@hotmail.com; 8 Department of Psychiatry & Behavioral Sciences, McGovern Medical School, University of Texas Health Science Center, Houston, TX 77054, USA

**Keywords:** Huntington’s disease, renin-angiotensin system, BACHD, angiotensin peptides

## Abstract

The Renin-Angiotensin System (RAS) is expressed in the central nervous system and has important functions that go beyond blood pressure regulation. Clinical and experimental studies have suggested that alterations in the brain RAS contribute to the development and progression of neurodegenerative diseases. However, there is limited information regarding the involvement of RAS components in Huntington’s disease (HD). Herein, we used the HD murine model, (BACHD), as well as samples from patients with HD to investigate the role of both the classical and alternative axes of RAS in HD pathophysiology. BACHD mice displayed worse motor performance in different behavioral tests alongside a decrease in the levels and activity of the components of the RAS alternative axis ACE2, Ang-(1-7), and Mas receptors in the striatum, prefrontal cortex, and hippocampus. BACHD mice also displayed a significant increase in mRNA expression of the AT1 receptor, a component of the RAS classical arm, in these key brain regions. Moreover, patients with manifest HD presented higher plasma levels of Ang-(1-7). No significant changes were found in the levels of ACE, ACE2, and Ang II. Our findings provided the first evidence that an imbalance in the RAS classical and counter-regulatory arms may play a role in HD pathophysiology.

## 1. Introduction

Huntington′s disease (HD) is an autosomal-dominant neurodegenerative disease caused by an unstable CAG triplet sequence expansion in the *huntingtin* gene *(Htt)*. This results in an abnormally long polyglutamine sequence in the N terminus of the huntingtin protein (HTT) [1,2]. In humans, the HTT gene usually contains 6 to 26 CAG repeats, whereas in HD, the gene presents more than 36 repeats resulting in a mutant HTT-induced disease [3,4].

Neuropathological characterization of HD reveals progressive loss of neurons in the striatum and cortex as well as in the hippocampus and hypothalamus [5,6]. Clinical manifestations of HD usually begin between 35 and 50 years of age and include motor symptoms along with cognitive and behavioral symptoms [7,8,9,10]. Cognitive and behavioral deficits can be detected in premanifest HD patients (i.e., before the clinical diagnosis of HD, which is based on motor symptoms), before deteriorating in later stages of the disease [11]. Currently, there are no effective disease-modifying treatments for HD [2]. New targets are needed for the development of therapeutic strategies.

The Renin-Angiotensin System (RAS) is well known for its role in the cardiovascular and renal systems, acting as a regulator of blood pressure and hydro-electrolyte homeostasis [12,13]. It is a complex endocrine system composed of a range of enzymes, peptides, and receptors [14]. The RAS has two main axes: the classical axis is mainly composed of the angiotensin-converting enzyme (ACE), angiotensin II (Ang II), and angiotensin type 1 (AT_1_) receptor; the counter-regulatory axis is formed by ACE2, Ang-(1-7), and the Mas receptor. The effects of Ang-(1-7) oppose the major responses induced by Ang II [12,14,15]. Apart from peripheral systems, the RAS components have also been found in the brain [16,17], prompting the investigation of the potential pathophysiological roles played by RAS in neuropsychiatric disorders [18,19,20,21,22,23,24,25] including HD [26,27].

Only a few studies have investigated the involvement of RAS components in HD. A decrease in ACE activity was described in different brain regions of HD patients such as the striatum, substantia nigra, nucleus accumbens, and caudate [26,28,29]. Conversely, increased ACE activity was found in the cerebrospinal fluid of HD patients [30]. Alterations in the levels of AT_1_ and AT_2_ receptors were also reported in brain homogenates of HD patients, suggesting the involvement of RAS classical axis components in HD pathophysiology [31]. Interestingly, HD patients exhibited higher serum levels of autoantibodies against the AT_1_ receptor compared with healthy controls, which were [32] associated with earlier disease onset and increased disease burden [33].

Herein, we took advantage of a well-established murine model of HD [34] and plasma from HD patients to investigate whether changes in the levels of molecules in the classical and alternative RAS arms may contribute to HD symptoms. The understanding of RAS components’ involvement in HD progression may open new avenues for the development of novel therapeutic strategies for HD.

## 2. Results

### 2.1. Motor and Behavioral Tests in Mice

**Figure 1:**
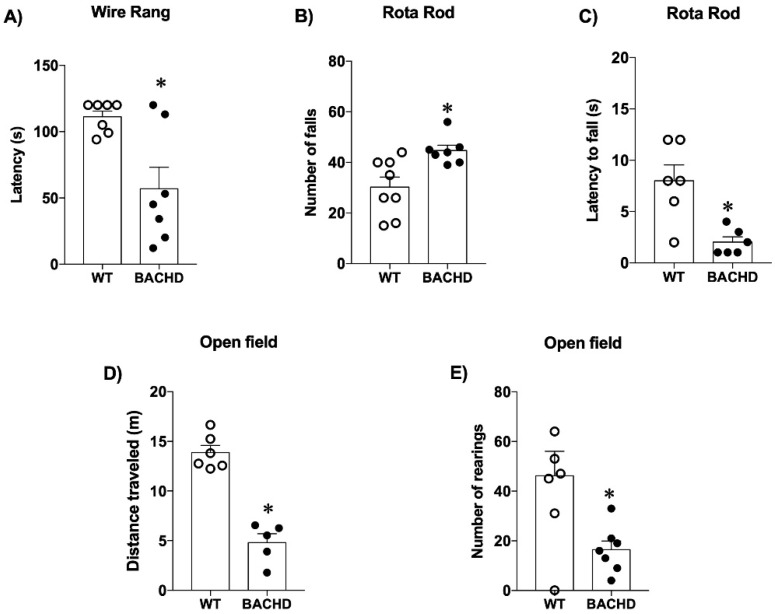
BACHD mice presented motor and behavioral changes. (**A**) BACHD mice showed impairment of muscle strength on the wire hang test. (**B**) BACHD mice exhibited an increased number of falls and (**C**) a decrease in the latency to fall in the rotarod test. (**D**) BACHD mice showed a decrease in the distance traveled on the open field and (**E**) a decreased number of rearings. Results are expressed as mean ± SEM. *n* = 5–7. * *p* < 0.05 compared with WT group. The data were analyzed by Student’s *t*-test.

To evaluate muscle strength, mice were submitted to the wire hang test. Statistical analysis revealed that BACHD mice show impaired muscle strength compared to WT mice [BACHD: t_(12)_ = 3.22; *p* = 0.0073] since they presented a decreased latency to fall (Figure 1A). Additionally, on the rotarod test, BACHD mice exhibited an increased number of falls [BACHD: t_(13)_ = 3.09; *p* = 0.0086] (Figure 1B) as well as a decrease in the latency to fall [BACHD: t_(10)_ = 3.67; *p* = 0.0043] (Figure 1C) compared with the WT group. Together, these results indicate that 24-month-old BACHD mice present impairments in motor learning, balance, and motor coordination. Ultimately, BACHD mice also presented decreased locomotor activity, as they travelled shorter distances [BACHD: t_(10)_ = 5.47; *p* = 0.0003] (Figure 1D) and performed less rearing than control mice [BACHD: t_(10)_ = 2.45; *p* = 0.0343] (Figure 1E) in the open field test.

### 2.2. Renin-Angiotensin System Components

**Figure 2:**
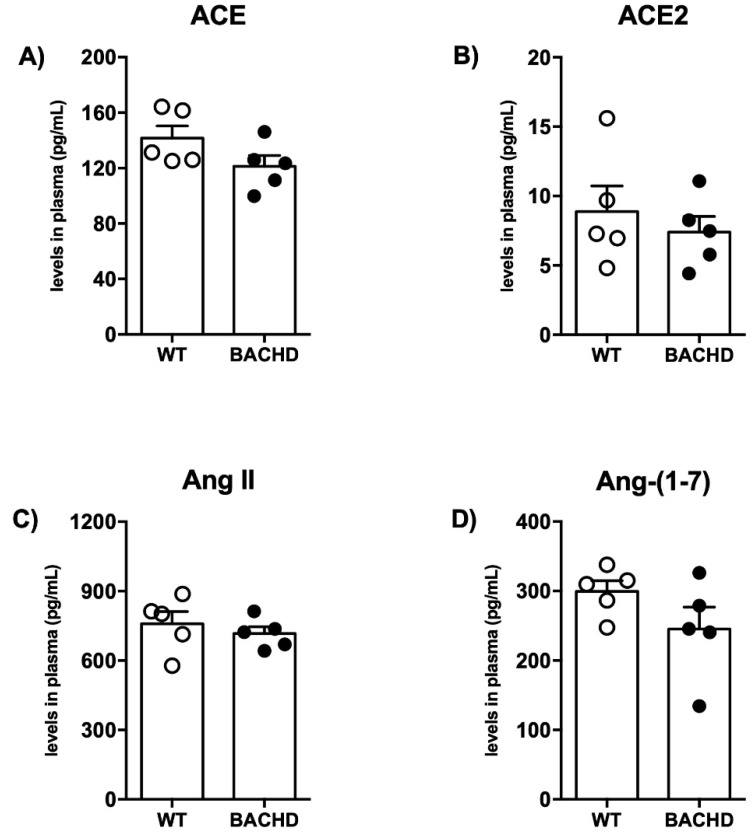
RAS components’ levels in the plasma of BACHD and WT mice. (**A**) ACE, (**B**) ACE2, (**C**) Ang II, and (**D**) Ang-(1-7) plasma levels are not altered in 24-month-old BACHD mice. Results are expressed as mean ± SEM. *n* = 5. The data were analyzed by Student’s *t*-test.

**Figure 3:**
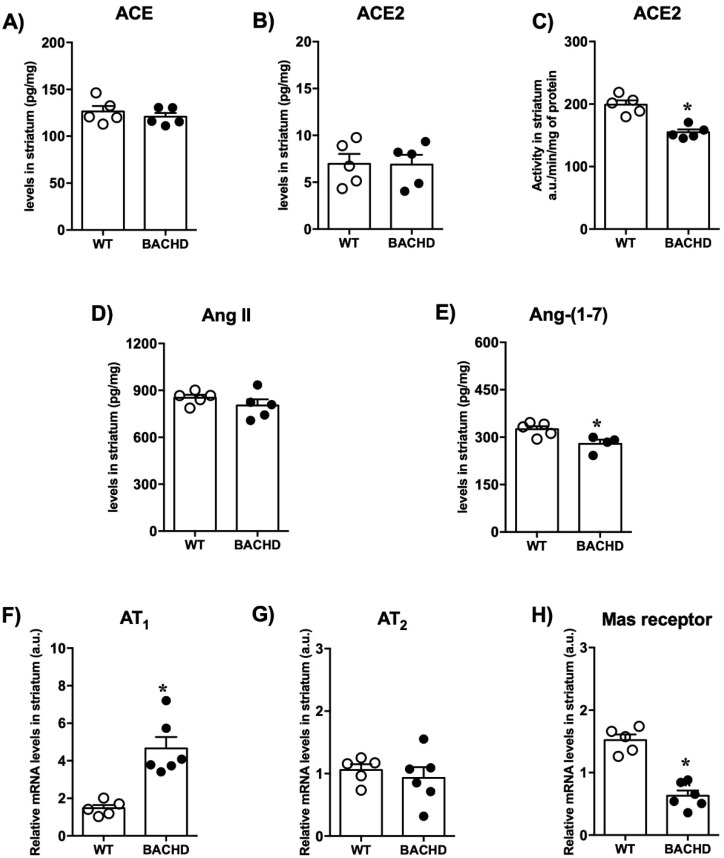
RAS components’ levels in the striatum of BACHD and WT mice. (**A**) ACE, (**B**) ACE2 levels, (**C**) ACE2 activity, (**D**) Ang II levels, (**E**) Ang-(1-7) levels, (**F**) AT_1_ receptor expression, (**G**) AT_2_ receptor expression, and (**H**) Mas receptor expression in the striatum of BACHD mice. Results are expressed as mean ± SEM. *n* = 5–6. * *p* < 0.05 compared with WT group. The data were analyzed by Student’s *t*-test.

As shown in Figure 2, no differences were found in the levels of ACE, Ang II, ACE2, and Ang-(1-7) in the plasma of BACHD mice compared with WT mice. However, when evaluating the components of RAS in the striatum, the main region affected by HD, we found interesting results. Although we did not find differences in ACE and ACE2 levels (Figure 3A,B), there was a reduction in the ACE2 activity in the striatum of BACHD mice (BACHD: 155 ± 4.5 vs. WT: 199 ± 6.8 a.u./min/mg of protein; *n* = 5) (Figure 3C). A reduction in Ang-(1-7) levels in the striatum of BACHD mice was also detected but no significant differences in the levels of Ang II (Figure 3D,E). In addition, there was increased mRNA expression for AT_1_ in BACHD animals compared with controls (BACHD: 4.7 ± 0.6 vs. WT: 1.5 ± 0.2 relative mRNA levels; *n* = 5–6) (Figure 3F). No significant changes were found in AT_2_ mRNA expression (Figure 3G). A significant decrease in Mas receptor mRNA expression was also observed in BACHD mice (BACHD: 0.6 ± 0.08 vs. WT:1.5 ± 0.09 relative mRNA levels; *n* = 5–6) (Figure 3H). 

**Figure 4:**
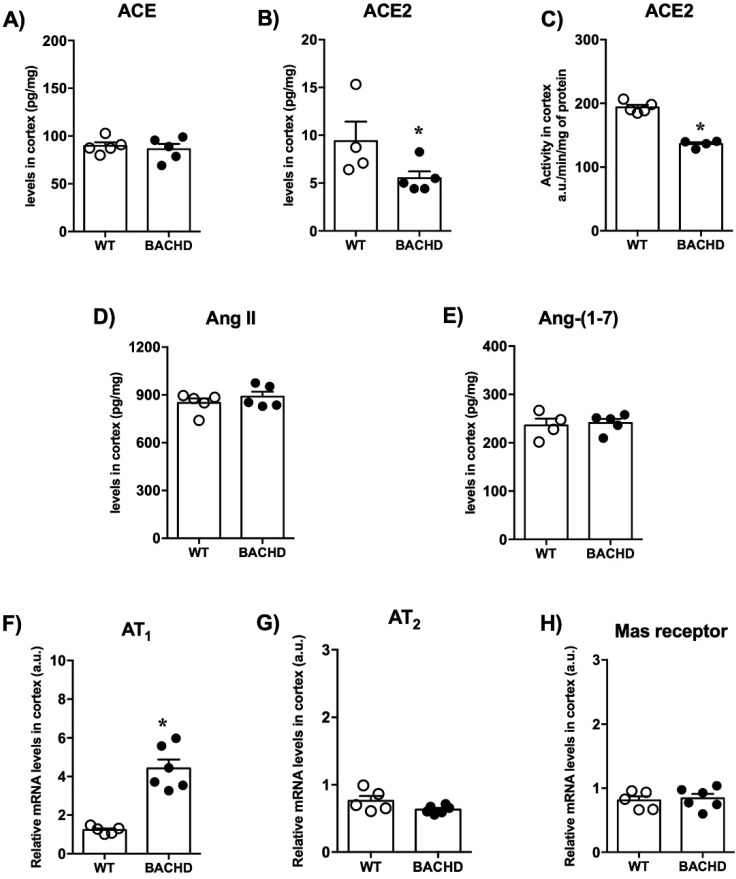
RAS components’ levels in the cerebral prefrontal cortex of BACHD and WT mice. (**A**) ACE, (**B**) ACE2 levels, (**C**) ACE2 activity, (**D**) Ang II levels, (**E**) Ang-(1-7) levels, (**F**) AT_1_ receptor expression, (**G**) AT_2_ receptor expression, and (**H**) Mas receptor expression in the cerebral cortex of BACHD mice. Results are expressed as mean ± SEM. *n* = 5–6. * *p* < 0.05 compared with WT group. The data were analyzed by Student’s *t*-test.

The expression of RAS components in the prefrontal cortex is shown in Figure 4. No alterations in ACE levels were found in the prefrontal cortex of BACHD mice compared with WT controls (Figure 4A). The levels and activity of ACE2 were significantly reduced in BACHD mice in comparison with WT mice (BACHD: 5.5 ± 0.7 vs. WT:9.4 ± 2.0 pg/mg; *n* = 5 and BACHD: 136 ± 2.8 vs. WT: 194 ± 4.0 a.u./min/mg of protein; *n* = 5) (Figure 4B,C). No significant differences in the levels of the peptides Ang II and Ang-(1-7) were found (Figure 4D,E). Interestingly, BACHD mice presented higher levels of AT_1_ receptor mRNA expression (4.4 ± 0.4 vs. 1.2 ± 0.08 relative mRNA levels; *n* = 5–6) (Figure 4F). Moreover, there were no significant differences in mRNA expression for AT_2_ and Mas receptors between the experimental groups (Figure 4G,H).

**Figure 5:**
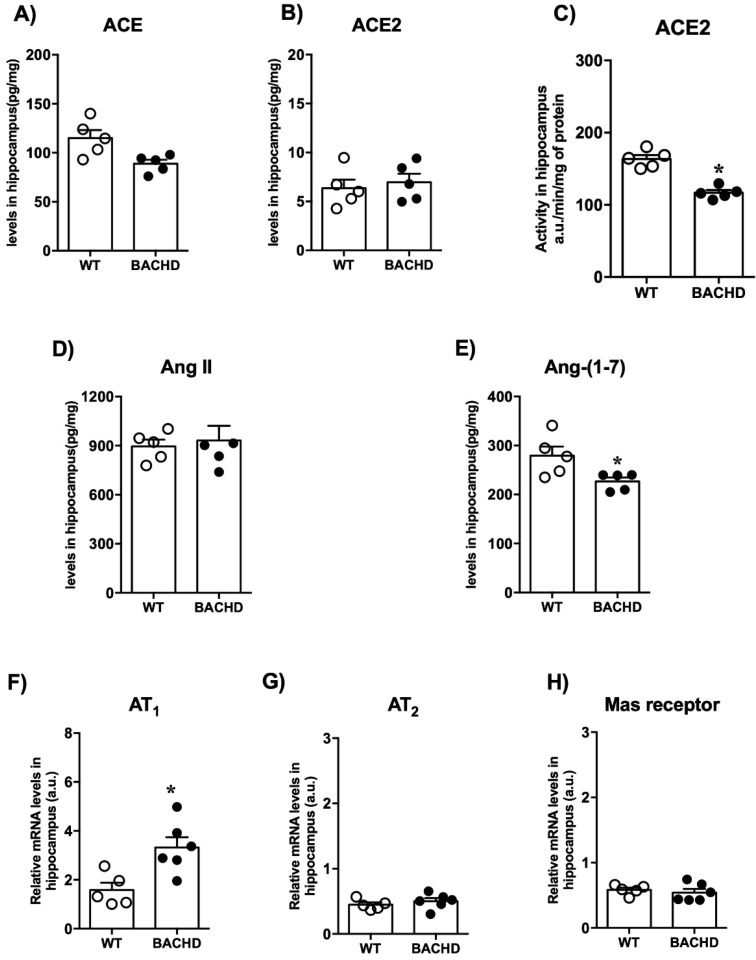
RAS components’ levels in the hippocampus of BACHD and WT mice. (**A**) ACE, (**B**) ACE2 levels, (**C**) ACE2 activity, (**D**) Ang II levels, (**E**) Ang-(1-7) levels, (**F**) AT_1_ receptor expression, (**G**) AT_2_ receptor expression, and (**H**) Mas receptor expression in the hippocampus of BACHD mice. Results are expressed as mean ± SEM. *n* = 5–6. * *p* < 0.05 compared with WT group. The data were analyzed by Student’s *t*-test.

We also evaluated the components of RAS in the hippocampus (Figure 5). We did not find differences in the ACE and ACE2 levels (Figure 5A,B). Interestingly, we found a reduction in ACE2 activity in the hippocampus of BACHD mice compared with controls (Figure 5C). Furthermore, BACHD and WT mice presented similar levels of Ang II (Figure 5D). When corroborating the results of the reduced ACE2 activity, we observed a reduction in Ang-(1-7) levels in BACHD animals (BACHD: 117 ± 3.8 vs. WT: 163 ± 5.4 a.u./min/mg of protein; *n* = 5) (Figure 5E). BACHD mice also presented higher levels of AT_1_ receptor mRNA expression compared with WT mice (BACHD: 3.3 ± 0.4 vs. WT: 1.6 ± 0.3 relative mRNA levels; *n* = 5–6) (Figure 5F). No significant differences in mRNA expression for AT_2_ and Mas receptors were found between the experimental groups (Figure 5F,G).

**Figure 6:**
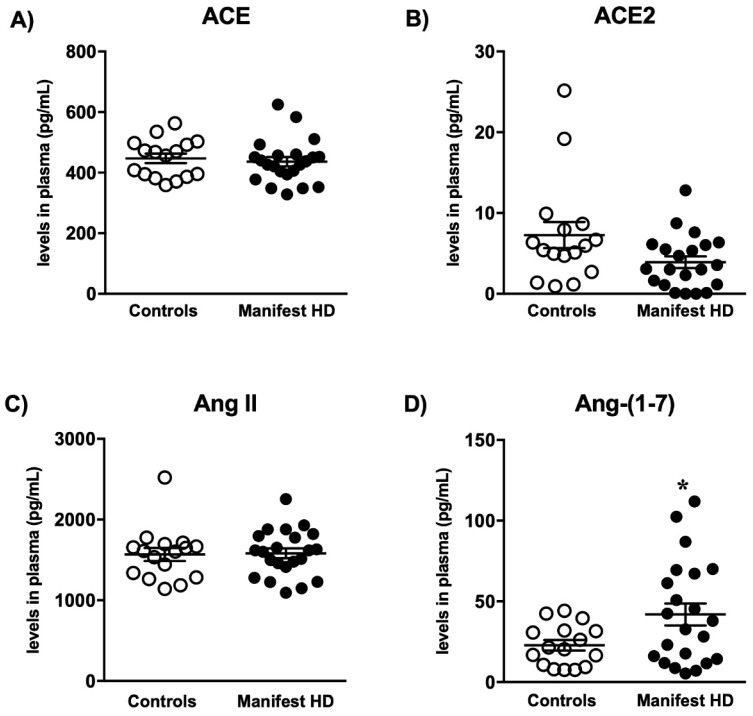
RAS components’ concentrations in the plasma of HD manifest and control patients. (**A**) ACE and (**B**) ACE2 levels, (**C**) Ang II and (**D**) Ang-(1-7) levels in the plasma of HD manifest and control patients. Results are expressed as mean ± SEM. *n* = 16–22. * *p* < 0.05 compared with the control group. The data were analyzed by Student’s *t*-test.

Lastly, we assessed the levels of RAS components in the plasma samples of patients with HD and healthy controls. The demographic and clinical data are shown in Table 1. The patients and controls did not differ in age, sex, and BMI. The groups presented similar levels in the components of the classical RAS axis, i.e., ACE and Ang II (Figure 6A,C). Interestingly, there were differences in the RAS counter-regulatory axis with significantly higher levels of Ang-(1-7) in the HD group (Figure 6D). No significant differences were found in the plasma levels of ACE2 (Figure 6B).

## 3. Discussion

In recent years, it has become evident that the RAS is active in several organs, including the brain, and has important functions that go beyond the cardiovascular and renal systems [13,16,17,35,36,37]. A growing body of evidence has supported the involvement of RAS in the development and progression of neurodegenerative conditions such as Parkinson’s disease (PD) and Alzheimer’s disease (AD) [20,21,22,23,24,35]. Taking advantage of a well-established murine model of HD as well as plasma samples of patients with HD, we provided novel evidence of an imbalance between the classical and the counter-regulatory RAS arms in HD. Our results were demonstrated in both local (mouse brain) and peripheral (plasma of patients) RAS.

The previous characterization of this transgenic model revealed progressive motor deficits in BACHD mice at 12 months old [34,38]. Our work, as far as we know, is the first to investigate how motor changes occur in BACHD animals at a more advanced age (24 months old), which may correspond to the HD endpoint. 

In parallel with motor impairment, the BACHD mice presented a significant reduction in the ACE2/Ang-(1-7)/Mas receptor axis in key brain areas associated with HD such as the striatum and hippocampus. Previous data regarding the role of RAS in HD is very limited and mainly focused on the classical axis components. It is worth mentioning that the animals used in the current study are 24 months old, corresponding to the HD endpoint. The evaluation of the RAS components in a later stage of the disease might explain, at least in part, the decrease in the levels of Ang-(1-7) in the striatum and the hippocampus of BACHD mice, culminating with significant motor impairment. The decrease in the concentration of Ang-(1-7) in the disease endpoint could suggest a lack of neuroprotective mechanisms. The improvement in cognitive decline following intracerebroventricular infusion of Ang-(1-7) in a mouse model of Alzheimer’s Disease [39], supports our findings.

Regarding the ACE/Ang II/AT1 receptor axis, we showed that the BACHD mice displayed enhanced mRNA expression of the AT_1_ receptor in all brain areas evaluated. Alterations in components of the RAS classical axis have been reported in brain samples of HD patients in comparison with controls [26,28,29]. Lower ACE activity was found in the striatum [26] as well as in the caudate nucleus [29] and substantia nigra [28] of HD patients. On the other hand, higher ACE activity was reported in the cerebrospinal fluid of HD patients compared with controls [30]. In addition, a study investigated the effects of Trandolapril, an ACE inhibitor, in an experimental model of HD induced by the infusion of 3-Nitropropionic acid (3-NP) in the striatum of rats. The administration of Trandolapril (4 and 6 mg/kg, p.o) daily for 12 days prevented motor and behavioral deficits and attenuated oxidative stress and mitochondrial enzyme activities in rat brains [40]. Finally, a study employing radioligand analysis revealed a 35% decrease in the levels of AT_1_ receptors in the putamen of HD patients in comparison with controls [31]. 

Herein, we also evaluated the levels of RAS components in the plasma of manifest HD gene carriers and controls. Patients with manifest HD presented higher circulating levels of Ang-(1-7) in comparison with controls, which might be a compensatory mechanism in an attempt to maintain the central nervous system homeostasis.

Although the pathophysiological significance of our findings is yet to be explained, they may support the scenario of ACE/Ang II/AT1 receptor axis activation and a counter-regulatory role of Ang-(1-7) in maintaining the brain function in response to neurodegenerative events. It has already been demonstrated that ACE2 plays a neurotrophic and protective role by activating the ACE2/Ang-(1-7)/Mas axis, inhibiting cognitive impairment in neurodegenerative diseases [41,42]. For instance, activation of the ACE2/Ang-(1-7)/Mas axis inhibited cognitive deficits in a rodent model of Alzheimer’s disease, potentially through its anti-apoptotic, anti-inflammatory, and neurotrophic effects [42]. Accordingly, cerebrospinal fluid levels of ACE were associated with an Amyloid-ß_42_ burden in patients diagnosed with Alzheimer’s Disease [43]. Finally, another study that investigated the circulating levels of RAS components in patients with Parkinson’s disease, also showed that lower circulating levels of Ang I, Ang II, and Ang-(1-7) were associated with increased severity of depressive symptoms [44].

A recent study from our workgroup demonstrated that despite the similar plasma levels of RAS components between controls, premanifest, and manifest HD gene carriers, there is a positive correlation between ACE2 levels and scores in the Verbal Fluency Test in HD. Thus, higher levels of ACE2 were associated with better verbal function. A negative correlation between Ang II levels and general cognition scores in the Mini-Mental State Examination was also found, indicating that higher concentrations of Ang II were associated with worse cognitive performance [45]. Taken together, these findings support a protective role of the counter-regulatory axis in HD. Further studies are necessary to better address whether RAS modulators that potentiate the ACE2 /Ang-(1-7) /Mas receptor axis and/or inhibit the ACE2 /AngII /AT_1_ receptor axis may improve HD-associated neuronal loss and symptoms.

In line with our findings, previous studies have demonstrated impairments in the ACE2/Ang-(1-7)/Mas receptor axis in other neurodegenerative diseases [18,21,39,46,47]. For instance, decreased levels of Ang-(1-7) were reported in the prefrontal cortex and hippocampus of senescence-accelerated mouse prone 8 (SAMP8) mice, a model for examining the pathophysiology of early changes in AD [48]. Importantly, an inverse correlation was also found between concentrations of Ang-(1-7) and tau hyperphosphorylation in those brain regions [48]. Similar findings were described in P301S mice, an animal model of tauopathy, supporting the role of the RAS alternative axis in neurodegenerative diseases [46]. In addition, intracerebroventricular infusion of Ang-(1-7) prevented cognitive decline and attenuated the hippocampal levels of phospho-tau and amyloid-beta peptide in other rodent models of AD [39,47]. Of note, the administration of A-779, an antagonist of the Mas receptor, hampered the beneficial effects of Ang-(1-7), indicating that the Ang-(1-7) protective activity was mediated by the activation of Mas receptors [47]. Corroborating these findings, the intraperitoneal injection of diminazene aceturate (DIZE), an ACE2 activator, improved cognitive impairment and synaptic and neuronal losses in the brains of SAMP8 mice [49]. Evidence from preclinical studies highlights the brain ACE2/Ang-(1-7)/Mas receptor axis as a potential target for the treatment of neurodegenerative diseases. In humans, a post-mortem study showed a reduction in ACE2 activity in the mid-frontal cortex of patients with AD compared with age-matched non-demented controls, corroborating, at least in part, our findings [21]. In addition, patients with AD had lower plasma levels of Ang-(1-7) than sex and age-matched controls and these levels were positively correlated with white matter lesions in magnetic resonance imaging [18].

Our findings provide original evidence that changes in the RAS classical and counter-regulatory axes may play a pathophysiological role in HD. Further studies are necessary to broaden our knowledge regarding the mechanisms underlying the RAS alternative arm’s protective effects and whether modulators of RAS might be a promising therapeutic strategy in HD.

## 4. Materials and Methods

### 4.1. Animal Model and Ethical Procedures

Our institutional committee that regulates the use of laboratory animals (Ethics Committee on Animal Experiments-CEUA/UFMG) approved all experimental protocols under protocol #036/2013. The FVB/NJ (wild-type-WT) and FVB/N-Tg (HTT * 97Q) IXwy/J (BACHD) transgenic mice (male) were held in a place with a controlled temperature (23 °C) in a 12–12h light–dark cycle. Food and water were provided ad libitum in an animal care facility at our institution. All animals used in this study were genotyped as previously described by [50]. In this study, we used 24-month-old WT and BACHD mice. We used 24-month-old BACHD animals, as these animals have established brain degeneration [34], allowing the investigation of potential RAS changes in the main brain areas affected by HD.

### 4.2. Open Field Test 

Spontaneous locomotor activity was assessed using an automatic open field apparatus (LE 8811 IR Motor Activity Monitors PANLAB, Harvard Apparatus; Barcelona, Spain), with acrylic box dimensions of 450 × 450 × 200 mm (width × depth × height). The open field was novel to the animals and the illumination in the room was 200 lx. Quantification of total activity was calculated using the ACTITRACK program (Panlab, Barcelona, Spain). The animals were placed in the center of the open field. The total distance traveled and the number of rearings were recorded during 10 min [51].

### 4.3. Rotarod

A rotarod [Insight, Ribeirão Preto, Brazil, 40 (w) × 30 (d) × 38 (h) cm; 16 r.p.m.] was used to evaluate motor coordination. In the rotarod experiments, mice were exposed to a pretest with 5 sessions of 2 min each. Animals that failed to equilibrate for 2 min in at least 1 of the 5 attempts were excluded from the experiment. After 24 h, the mice that completed the previous task were exposed again to the apparatus. Latency to fall from the rotating cylinder was recorded. The time limit for mice to remain on the rotarod was up to 300 s [52,53]. 

### 4.4. Wire Hang Test

The wire hang test was used to assess balance and muscle strength. This apparatus consists of a box (10 × 10 × 10 cm) and a wire mesh grid on its top. To evaluate muscle strength, the animals were placed on the wire mesh, which was then inverted so that the animal gripped the wire. Latency to fall was recorded with a 120 s cut-off time [54].

### 4.5. Measurement of RAS Components

Key brain regions such as the prefrontal cortex, hippocampus, and striatum were carefully dissected and then homogenized in an extraction solution (100 mg of tissue per milliliter), containing 0.4 M NaCl, 0.05% Tween 20, 0.5% BSA, 0.1 mM phenylmethylsulphonyl fluoride, 0.1 mM benzethonium chloride, 10 mM EDTA, and 20 KIU aprotinin, using Ultra-Turrax (IKA®-Werke GmbH & CO, Staufen, Alemanha). Lysates were centrifuged at 13,000× *g* for 10 min at 4 °C and supernatants were collected. Samples were then thawed and the tissue levels of ACE, Ang II, ACE2, and Ang-(1-7) were measured by ELISA, according to the procedures supplied by the manufacturer (MyBioSource, San Diego, CA, USA). All kits applied the sandwich ELISA technique, except for the ACE measurement, whose kit applied the competitive ELISA method. The sensitivity of the assay was 10 pg/mL for ACE and ACE2; 12 pg/mL for Ang II; and 1.5 pg/mL for Ang-(1-7). 

### 4.6. Measurement of ACE2 Activity

The enzymatic activity of ACE2 was determined in the striatum, prefrontal cortex, and hippocampus homogenates using a fluorogenic substrate (fluorogenic peptide VI; R & D Systems, Minneapolis, MN, USA). The enzymatic activity was measured using the SpectraMax Gemini EM Fluorescence Reader (Molecular Devices, San Jose, CA, USA), as previously described [55,56]. Samples were read every minute for 60 min, beginning immediately after the addition of the fluorogenic peptide substrate at 37 °C. The results of each sample were expressed as arbitrary units (a.u.) corresponding to the average of the last 5 min of readings when the reaction reached the plateau, corrected for mg of protein, which was measured by the Bradford method. 

### 4.7. Measurement of RAS Receptors’ mRNA Expression 

The striatum, prefrontal cortex, and hippocampus samples were collected immediately on dry ice and stored at −80 °C to prevent RNA degradation. Total RNA isolation was performed following the Trizol reagent method (Invitrogen, Life Technologies, Waltham, MA, USA) according to the manufacturer’s protocol. RNA samples (2 µg) were treated with DNase to eliminate genomic DNA present in the samples. The mRNA expression was assessed by qRT-PCR after reverse transcription with Moloney murine leukemia virus (MML-V) (Invitrogen Life Technologies, Waltham, MA, USA). The cDNA for endogenous S26 ribosomal (endogenous control) and AT_1_, AT_2,_ and Mas receptors were amplified using specific primers and SYBR Green reagent(Applied Biosystems, Waltham, MA, USA). The reactions were performed using 40 cycles and an annealing temperature of 60 °C (ABI Prism 7000, Applied Biosystem, Waltham, MA, USA). The gene expression was quantified using the comparative threshold cycle (Ct) method. Primer sequences were AT_1_: 5’-GGT GGG AAT ATT GGA AAC AG-3’ (forward) and 5’-AAG AAG AAA AGC ACA ATC GCC-3’ (reverse); AT_2_: 5’-GCT GAG TAA GCT GAT TTA TG-3’ (forward) and 5’TTA AGA CAC AAA GGT GTC CA-3’ (reverse); Mas receptor: 5’-CCC ACC CAT TCC CAT AGT GC-3’ (forward) and 5’-CCG AGA GGA GAG ATG CTC ATG-3’ (reverse); and the endogenous control S26: 5’-CGA TTC CTG ACA ACC TTG CTA TG-3’ (forward) and 5’-CGT GCT TCC CAA GCT CTA TGT-3’ (reverse).

### 4.8. Assessment of Plasma Levels of Proteins Related to the RAS in Human Samples

Herein, we included 22 patients with a genetic and clinical diagnosis of HD (manifest HD) and a group of 16 controls with comparable age, sex, and body mass index (BMI). The genetic diagnosis was confirmed by a genotype larger CAG allele ≥ 36. The clinical HD diagnosis was based on the motor signs certainty, i.e., a Diagnostic Confidence Level (DCL) set to 4 in the Unified HD Rating Scale (UHDRS) (1). Patients were recruited from the Huntington’s Disease Society of America (HDSA) Center of Excellence at the University of Texas Health Science Center in Houston (UTHealth). Controls were recruited from the local community, comprising a group of people with no history of neurological or psychiatric disorders. All subjects provided written informed consent before admission to the study. The Research Ethics Committee of UTHealth approved this study.

Ten milliliters of blood were drawn by venipuncture in vacuum tubes containing heparin, on the same day of the clinical assessment. Blood was collected in a non-fasting state. Whole blood samples collected were used for plasma obtaining within two hours of having been drawn. These samples were centrifuged twice at 3000× *g* for 10 min at a temperature of 4 °C. Plasma was collected and stored at −80 °C until assayed. Samples were then thawed and plasma levels of Ang II, Ang-(1-7), ACE, and ACE2 were measured by ELISA according to the procedures supplied by the manufacturer (MyBioSource, San Diego, CA, USA). All kits applied the sandwich ELISA technique, except for the ACE measurement, whose kit applied the competitive ELISA method. Concentrations were expressed as pg/mL. The sensitivity of the assays was 1.0 pg/mL for ACE and ACE2; 2.0 pg/mL for Ang-(1-7); and 18.75 pg/mL for Ang II.

### 4.9. Statistical Analysis 

All values of animal protocols were expressed as mean ± standard error of the mean (SEM). Differences among groups were assessed by Student’s t-test. For the protocols using human samples, the statistical analysis was performed as follows: Association between dichotomous variables was assessed with Fisher’s exact test. All variables were tested for Gaussian distribution using the Kolmogorov–Smirnov normality test. Comparisons between patients and controls were made using the Student’s *t*-test, when the data were determined to follow a normal distribution, or the Mann–Whitney test when the variables did not follow a normal distribution. 

All statistical tests were two-tailed and were performed using a significance level of α = 0.05. Statistical analyses were performed using SPSS software version 25.0 (SPSS Inc., Chicago, IL, USA) as well as GraphPad Prism version 6.0 (GraphPad Software, Inc., La Jolla, CA, USA).

## Figures and Tables

**Table 1:** Demographic and clinical data of patients with HD and controls.

	Controls (*n* = 16)	Manifest HD (*n* = 22)	*p*-Value
Age in years	48.12 ± 11.02 (47.5)	49.88 ± 12.64 (49.4)	0.661 ^a^
Female sex, *n* (%)	10 (62.5)	14 (66.7)	1.000 ^b^
BMI (Kg/m^2^)	28.51 ± 7.32 (25)	27.62 ± 6.48 (25)	0.650 ^c^
CAG length (larger allele)	-	44.63 ± 4.14 (43)	-
UHDRS TMS	-	29.67 ± 12.91 (30)	-
TFC score	-	8.43 ± 2.82 (8)	-
Independence Scale (%)	-	84.52 ± 12.14 (85)	-
Ang II (pg/mL)	1567.77 ± 325.19 (1608)	1598.50 ± 282.83 (1618)	0.761 ^a^
ACE (pg/mL)	447.21 ± 62.69 (463)	435.49 ± 73.94 (426)	0.614 ^a^
Ang-(1-7) (pg/mL)	22.80 ± 12.80 (21)	39.78 ± 31.04 (33)	0.048 ^a^
ACE2 (pg/mL)	7.27 ± 6.46 (5.7)	6.05 ± 7.86 (4.7)	0.189 ^c^

Abbreviations: BMI = body mass index; HD = Huntington’s disease; TFC = UHDRS = Unified HD Rating Scale. Values are given as mean ± SD (median). ^a^ Student’s *t*-test; ^b^ Fisher’s exact test; ^c^ Mann–Whitney test.

## Data Availability

The datasets generated during and/or analyzed during the current study are not publicly available due to [NOT PUBLIC] but are available from the corresponding author on reasonable request.
